# Clerical Mortuary

**Published:** 1856-03

**Authors:** 


### CLERICAL MORTUARY.

Of Clergymen of all denominations, dying during 1855, in the United States, there were one hundred and twenty. The smallest number, five, died in February; the largest number, seventeen, died in October. The three most healthful consecutive months, were December, January and February, giving twenty-two deaths, or about one-fifth of the whole; the three most fatal consecutive months, were September, October and November, giving thirty-nine deaths, or one-thirth of the whole; showing the error of the prevalent opinion, that "bad weather," as it is called, is unhealthy, necessarily; for, during the most inclement months of the year, the smallest number died; while, during the three fall months, when the weather is neither too cool nor too hot, the mortality is nearly double. Railroad conductors, who are in and out of suffocating cars incessantly during the coldest months of the year, are observably healthy men. The men of the Arctic Expeditions do not die of bad colds, pleurisies, and the like. Persons often make the inquiry, when in a decline, *will it hurt me to go out of doors?* Our almost universal reply is, "No! it will do you good. Go out, rain or shine; if it is raining, have an umbrella, and let it rain on." How is it? Part of the lungs are gone, or at least they are working imperfectly, consequently such person is living on a less amount of air than the system requires; hence, the air he does consume should be the purest possible; and as no air within any four walls can be pure, the air of out doors, during daylight, must be the most proper for all, especially for consumptives, the world over. It is the irrational dread of *taking cold*, by going out of doors, which kills nine consumptives out of ten, far sooner than the disease itself would have done. If any man, sick or well, wants an infallible receipt for getting into that unfortunate condition in which "the slightest thing in the world gives him a cold," let him hover around the fire all day, let him bundle up, head and ears, every time he puts his head out at a door or window, and besides, keep his room regulated to a degree, for months at a time. Such a person never can get well of any thing; such a person, with such habits persevered in, will die, long before his time, it matters not what may be his ailment. Under "*the sunny skies of Italy*," where, according to poetic account, it is a happiness to breathe, so balmy is its atmosphere, the average of human life is shorter than in any other civilized country. Do not fear then the bleak December or the fiercer January, unless quite an invalid, or very old; the first consideration with the infant of one year or seventy-five, is *warmth*, *warmth*, WARMTH.

There is, however, one condition of the weather which all, except those in good health, should endeavor to avoid. An *East wind* is as fraught with danger and calamity now, as it was in the days of Scripture history. Such winds prevail after rains in this country, and there is a rawness and a dampness about them, which urgently calls for shelter for man and beast, even in mid summer. None, not even the healthy, can be exposed to east or northeast winds in the United States, at least, east of the Rocky Mountains, with impunity, except under one condition, and that is, under circumstances of bodily activity, sufficient to keep off all feeling of chilliness, and when such activity ceases, immediate retirement to a closed room, if indeed not to a good fire, even if in summer time, for it is in summer time the most consumptive colds originate.

Of the 120 clergymen dying during 1855, two-thirds, eighty, have their ages recorded, the youngest 27, the oldest 94; of these eighty, ONE-HALF had passed "three score and ten," thus confirming the generally received opinion of staticians that *Theologians are the longest lived of all the members of the human family*, the reasons for which, we believe, are mainly these:

1st. Being poorly supported, they have to "*rough it*;" the luxuries of life are impossible to them.

2d. The largest portion of their time, as a class, is spent on horseback, or other modes of travel, thus securing a large consumption of out-door air, with the very great advantage of frequent changes of air, food, and mode of preparation.

3. Pleasurable associations. The contemplations of a minister, are of a soothing character; his is a mission of love, of pure benevolence, the exercise of which, must always be happyfying.

Not only so, the clergymen of this country, and we feel thankful that it is so, are everywhere received with a respectful, cordial and affectionate welcome. What house is there in this whole land, outside of cities, where every thing is upside down, wrong end foremost, antipodean, except in materiel benevolences, where, we say, can a family be found, which has not at least one *Martha* to be careful of the minister's comfort, that he have the best of every thing; and in return for these attentions, aside from duty and natural solicitude for their spiritual welfare, there runs out from the minister's heart towards those with whom he is brought in contact, a living stream of tender concern, which in its reflex influences, gives warmth and health to soul and body; thus verifying the promise that those who love and serve God best, not only have *the life that now is, but that which is to come.* Having secured religion, all other necessary things are thrown in.

